# Cerebral Toxoplasmosis Mimicking a Brain Neoplasm in an Inaugural HIV-Positive Patient: The Importance of Early Decision-Making and Background Assessment in the Emergency Department

**DOI:** 10.7759/cureus.76936

**Published:** 2025-01-05

**Authors:** Diogo Alves, Patrícia Sobrosa, Rita Morais Passos, Francisco Silva, António Ferreira, Rogério Corga da Silva, Duarte Silva

**Affiliations:** 1 Critical Care, Unidade Local de Saúde do Alto Minho, Viana do Castelo, PRT; 2 Internal Medicine, Unidade Local de Saúde do Alto Minho, Viana do Castelo, PRT; 3 Critical Care, Hospital de Viana do Castelo, Viana do Castelo, PRT

**Keywords:** cerebral toxoplasmosis, decision-making in critical care, ethical concerns, hiv, neuroimaging

## Abstract

Intracranial lesions can present a diagnostic challenge in patients without previously known immunosuppression. When focal neurological signs and seizures occur in a patient with no established medical history, an expansive brain lesion may be initially interpreted as a neoplasm, influencing early clinical decisions regarding the extent of supportive measures. However, opportunistic infections, such as cerebral toxoplasmosis, should remain on the differential diagnosis - particularly after consideration of the patient’s background and potential epidemiological risks.

We present the case of a middle-aged woman of African origin who presented with new-onset seizures and a prolonged history of anorexia and weight loss. Initial neuroimaging suggested a primary or metastatic brain tumor, raising concerns regarding the patient's prognosis and the appropriateness of aggressive support in the emergency setting. The patient received corticosteroids and anticonvulsants in the emergency department (ED), with a subsequent need to start noninvasive ventilation.

Further laboratory workup revealed the inaugural human immunodeficiency virus (HIV) and *Toxoplasma gondii* infection rather than a neoplastic process. Following targeted antimicrobial therapy and initiation of antiretroviral treatment (ART), she demonstrated remarkable neurological and functional recovery. This case underscores the importance of maintaining a broad differential diagnosis in the ED, performing a thorough background evaluation of patients, and sustaining supportive management until a definitive diagnosis is established.

## Introduction

The evaluation of intracranial lesions frequently begins with a broad differential diagnosis. Malignancy constitutes a primary concern, particularly when imaging reveals findings such as mass effect, contrast enhancement, or perilesional edema. Non-neoplastic conditions can closely mimic these radiological features, posing significant diagnostic challenges. The ambiguous clinical presentation and overlapping imaging characteristics further enhance the challenge. Differential diagnoses for such lesions include demyelinating, vascular, inflammatory, and infectious diseases, but the proportion of patients who ultimately have a non-oncologic diagnosis is unknown [1].

Early and accurate differentiation between infectious and neoplastic causes of central nervous system (CNS) lesions is crucial for guiding appropriate management and reducing morbidity and mortality. Misclassifying an infectious lesion as malignant can delay life-saving antimicrobial or antiparasitic therapy, while pursuing invasive interventions can lead to additional morbidity [2]. One example is toxoplasmosis encephalitis (TE), where prompt treatment can result in dramatic clinical improvement. Studies show a 71% response to therapy, with the vast majority demonstrating ≥50% improvement in their baseline abnormalities by day 14 of therapy [3].

TE is the most common CNS infection in patients with advanced HIV, particularly those with CD4+ counts below 200 cells/μL who are not receiving effective prophylaxis. Clinically, TE typically presents with headache, confusion, and fever, alongside focal neurologic deficits or seizures. Mental status changes range from dull affect to stupor and coma and can result from global encephalitis or increased intracranial pressure [4]. On imaging, multiple ring-enhancing lesions surrounded by vasogenic edema are common, often localized to the basal ganglia, although other regions may also be involved [4,5]. These findings, while suggestive, overlap significantly with other pathologies, such as primary CNS lymphoma (PCNSL), bacterial abscesses, and even glioblastoma, complicating the diagnostic process. Magnetic resonance imaging (MRI), the preferred imaging modality, is more sensitive than computerized tomography (CT) for detecting these lesions; however, it still lacks the specificity needed to distinguish TE from other CNS lesions and is not widely or promptly available [5].

This diagnostic ambiguity prompts a need for comprehensive evaluation, integrating patient history, epidemiological context, and diagnostic testing for HIV and *Toxoplasma gondii *antibodies [6].

This report highlights the case of a patient with suspected malignancy based on imaging findings, later diagnosed with TE. The case underscores the importance of integrating clinical data, epidemiological context, and diagnostic testing in evaluating CNS lesions.

## Case presentation

Initial presentation

A previously healthy, 59-year-old woman of African origin was brought to the ED by her son after experiencing intense headaches for a few days and becoming unresponsive since that morning. She had arrived in Portugal, coming from São Tomé and Príncipe, just a few weeks prior. According to her family, she had been experiencing progressive anorexia and unintentional weight loss of more than 20 kg over the preceding three months, going from 60 kg to 40 kg of total body weight (33% of total body weight lost), and started needing help performing daily activities. No history of fever or other symptoms was apparent. Despite her deteriorating nutritional status, she had not sought medical evaluation before this acute event.

Initial ED evaluation and management

At the first medical examination at the ED, she presented with a Glasgow Coma Scale (GCS) of 10 (E3V2M5). She was tachycardic (heart rate: 112 bpm), normotensive (blood pressure: 125/75 mmHg), bradypneic (respiratory rate of six cycles per minute), with oxygen saturation of 85% on room air. Arterial blood gas analysis revealed hypoxemia (pO_2_ of 57 mmHg), and respiratory acidosis (pCO_2_ of 73 mmHg) with acidemia (pH of 7.21), prompting immediate concern for respiratory compromise. She was started on non-invasive ventilation (NIV) to correct the respiratory acidosis. Further workup revealed mild normochromic, normocytic anemia (hemoglobin: 9.2 g/dL), alongside normal renal and hepatic function. No overt leukocytosis (6.62 × 10³/μL) nor elevated inflammatory markers (C-reactive protein: 0.50 mg/dL) were identified, but hyponatremia (126 mmol/L) was noted (Table 1). Viral polymerase chain reaction (PCR) panel for respiratory virus and urine drug screening were negative.

**Table 1:** Relevant parameters at hospital admission pCO_2_: partial pressure of carbon dioxide; pO_2_: partial pressure of oxygen; WBC: white blood cells

Parameter	Value	Reference range
Hemoglobin (g/dL)	9.2	11.0-16.0
Total WBC count (x10^3^/µl)	6.620	4.000-10.000
C-reactive protein (mg/dL)	0.50	<0.51
Sodium (mmol/L)	126	136-145
Arterial pH	7.21	7.35-7.45
Arterial pCO_2_ (mmHg)	73	35-45
Arterial pO_2_ (mmHg)	57	80-100

**Figure 1:**
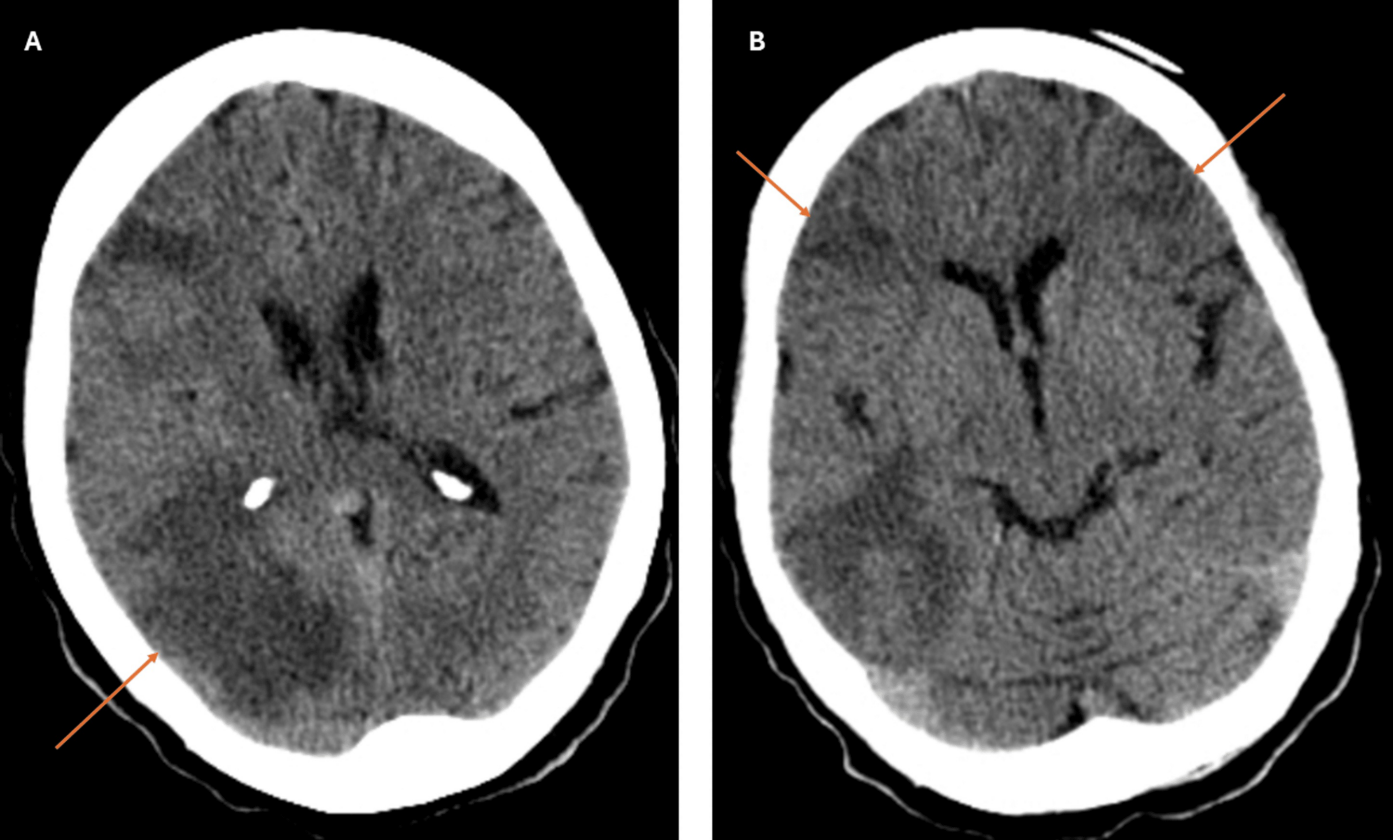
Images of brain CT scan Brain CT scan at hospital admission showing a hypodense lesion involving the right parieto-occipital region (arrow) with a predominant cortico-subcortical edematous component, associated with local sulcal effacement and right lateral ventricle collapse (A). Additional smaller lesions with edematous components (arrows) were noted bilaterally in the frontal regions (B).

A non-contrast head CT demonstrated a hypodense lesion involving the right parieto-occipital region with a predominant cortico-subcortical edematous component, associated with local sulcal effacement and right lateral ventricle collapse (Figure 1A). Additional smaller lesions with edematous components were noted bilaterally in the frontal regions (Figure 1B). Given the multiplicity and characteristics of the lesions, the report considered secondary deposits as the most likely possibility.

Consulting with neurosurgery raised the hypothesis of a secondary metastatic lesion or a PCNSL with associated inflammation and edema. Treatment with intravenous dexamethasone 10 mg was started.

While returning from the radiology department, the patient had a generalized tonic-clonic seizure requiring the administration of intravenous diazepam 10 mg and initiation of intravenous levetiracetam 1500 mg. After seizure termination, her GCS deteriorated to 8 (E2V2M4) in the postictal phase.

The medical team engaged in complex discussions about the diagnostic challenges posed by the patient’s condition. The primary debate centered on the utility and the appropriateness of aggressive interventions, such as orotracheal intubation, in a cachectic patient with a Clinical Frailty Scale of 6. Without a definitive diagnosis, the team faced a critical decision: whether to commit to invasive support and evaluation or consider early palliative measures, given the presumed malignant lesion and poor prognosis.

An urgent whole-body CT scan was performed to identify a possible primary lesion. The scan revealed no other lesions, supporting the decision to proceed with escalation of care.

Despite her poor mental status, the decision was made to delay intubation and continue NIV, critical to stabilize her respiratory function and prevent further neurological deterioration due to hypercapnia. Her vital signs were within acceptable limits, her respiratory status improved with NIV, and her seizures were controlled. She was closely monitored for 48 hours with continuous cardiorespiratory monitoring and neurological assessment. Medicated with seizure prophylaxis with intravenous levetiracetam 1500 mg every 12 hours, cerebral edema control with intravenous dexamethasone 4 mg every six hours, intravenous fluids, and careful electrolyte management. This approach proved effective, as her respiratory and neurological status began improving. NIV was weaned successfully, and hyponatremia was corrected with mental status slowly recovering to a GCS of 14 (E4V4M6).

During the first day, a comprehensive study was made to search for a correct diagnosis. Her recent arrival from a region in Africa with a higher prevalence of HIV raised suspicion of an underlying immunocompromised state. HIV testing confirmed positive for HIV-1, opening a myriad of potential causes for the cerebral lesions, including various infectious etiologies.

This led to an extensive diagnostic workup that included a broad range of infectious and inflammatory markers. Tests included immunoglobulins (A, G, and M), CD4+ count, venereal disease research laboratory (VDRL), cryptococcal antigen testing, toxoplasma, cytomegalovirus (CMV), and Epstein-Barr virus (EBV) serology, as well as bacterial, fungal, and mycobacterial blood cultures (Table 2). A lumbar puncture was executed, and cerebrospinal fluid (CSF) was analyzed for biochemical markers and PCR tests for toxoplasma, John Cunningham (JC) virus, EBV, BK virus, and CMV. Bacterial, fungal, and mycobacterial cultures were performed in CSF (Table 3).

**Table 2:** Infectious disease blood serum workup results HBs: hepatitis B surface; HCV: hepatitis C virus; HIV: human immunodeficiency virus; Ig: immunoglobulin; VDRL: venereal disease research laboratory test

Parameter	Value	Reference range
IgA (mg/dL)	608.0	60-400
IgG (mg/dL)	1953.0	700-1600
IgM (mg/dL)	399.0	40-230
Antigen/antibody ΗІV-1/2 immunoassay	Reactive	-
HIV-1/HІV-2 antibody differentiation immunoassay	Positive for HIV-1, Negative for HIV-2	-
HIV-1 viral load (Copys/mL)	895907	<20
Absolute CD4+ count (Cells/µL)	24	404-1612
VDRL	Negative	-
Antigen HBs (S/CO)	Non-reactive (0.52)	Reactive: >1
Total HCV antibodies	Negative	-
*Cryptococcus neoformans/Cryptococcus gatti* antigen	Negative	-
*Toxoplasma gondii* antibodies (UI/mL)	IgM - Non-reactive (0.08)	Reactive: >1.6
	IgG - Reactive (157.8)	Reactive: >3
Cytomegalovirus antibodies (AU/mL)	IgM - Non-reactive (0.08)	Reactive >1.0
	IgG - Reactive (233.5)	Reactive: >6
Epstein-Barr virus antibodies (S/CO)	IgM - Non-reactive (0.05)	Reactive: >1
	IgG - Reactive (68.6)	Reactive: >1
Blood cultures	Negative	-

**Table 3:** Cerebrospinal fluid workup results ADA: adenosine deaminase; CSF: cerebrospinal fluid; PCR: polymerase chain reaction; JC: John Cunningham; CMV: cytomegalovirus

Parameter	Value	Reference range
Cell count (cells/mm^3^)	0	0-5
Glucose (mg/dL)	65	40-70
ADA (U/L)	1.3	<9
Proteins (mg/dL)	124.30	15-45
CSF cultures	Negative	-
PCR JC virus	Negative	-
PCR Epstein-Barr virus	Negative	-
PCR *Toxoplasma gondii*	Positive	-
PCR CMV	Negative	-
PCR *Mycobacterium tuberculosis*	Negative	-
India ink staining	Negative	-
*Cryptococcus neoformans*/*Cryptococcus gatti *antigen	Negative	-

Subsequent workup on the general ward

**Figure 2:**
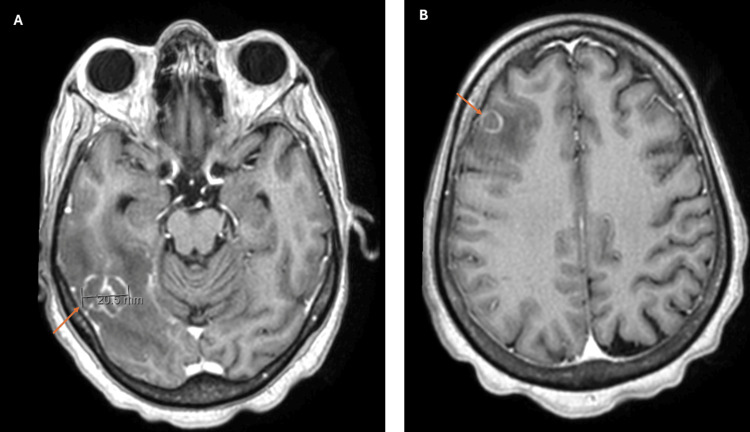
Brain MRI scan Two axial T1-weighted images (A and B) of brain MRI showing multiple ring-enhancing lesions (arrows). The frontal lesion has sub-centimetric diameter (B), and the lesion in the inferior and posterior right temporal region measures approximately 2 cm (A).

**Figure 3:**
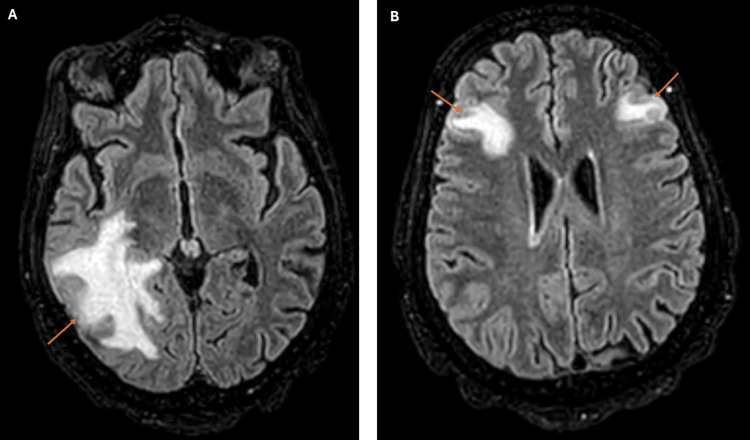
Brain MRI scan Two FLAIR images (A and B) of brain MRI showing areas of edema (arrows) in the right temporo-occipital (A) and bilateral frontal regions (B), causing sulcal effacement and locoregional mass effect. FLAIR: fluid-attenuated inversion recovery

On day 3, the patient was stable enough to be transferred to a general medical ward for further evaluation. A CD4+ T-cell count was found to be severely depressed (24 cells/µL), and serologic evaluation revealed positive for *T. gondii* IgG, providing a crucial clue that this lesion could be due to cerebral toxoplasmosis. Trimethoprim-sulfamethoxazole double-strength tablet daily was initiated as first-line therapy. Despite the recommendation for immediate initiation of ART in the presence of HIV and opportunistic diseases like TE, due to extensive brain edema and risk of immune reconstitution inflammatory syndrome, ART was delayed until documentation of edema reduction. An MRI of the brain with gadolinium contrast revealed multiple ring-enhancing lesions (Figure 2) with surrounding edema (Figure 3), findings that couldn’t rule out the suspicion of neoplasm but were more in favor of an infectious etiology. A few days later, clinical improvement and detection of *T. gondii* DNA in CSF confirmed the diagnosis.

The patient showed a notable improvement in her mental status; by day 10 of admission, she became more talkative, recognized family members, and followed commands. Repeat MRI after two weeks of therapy demonstrated a substantial reduction in the size and edema of the intracranial lesion. At this time ART was initiated with bictegravir 50 mg, emtricitabine 200 mg, and tenofovir 25 mg once daily, without adverse reactions. Over the ensuing days, her mobility improved, and she could ambulate with support. Levetiracetam and dexamethasone were weaned successfully without additional seizure activity. Ultimately, the patient regained near-normal neurological function and was discharged home with outpatient follow-up.

## Discussion

In Western countries, the most common causes of CNS lesions in immunocompetent patients are metastatic brain tumors and primary brain neoplasms. Metastatic tumors, which often originate from lung cancer, melanoma, or breast cancer, become increasingly prevalent in adults over the age of 30 to 40 years and account for more than half of all brain tumors in this population​ [7,8]. Meningiomas and glial tumors (e.g., glioblastoma, astrocytoma, oligodendroglioma) account for approximately two-thirds of all primary brain tumors [7]. However, in patients with HIV, the differential diagnosis must also include opportunistic infections such as TE, which is the most common CNS infection in this population [4].

The prognosis for patients with brain neoplasms and those classified as highly frail is consistently poor, often marked by high mortality rates and limited survival outcomes [8,9]. In contrast, patients with HIV infection exhibit short-term outcomes primarily determined by the severity of the acute illness and preadmission health status, including functional capacity and weight loss [10]. These factors suggest that ICU support for HIV patients is not inherently futile. However, such outcomes raise significant ethical and practical concerns regarding the pursuit of intensive care in these vulnerable populations. In our case, a challenging decision about ICU support had to be made with incomplete data. On one hand, the patient exhibited a high frailty level due to prolonged disease and an initial working diagnosis of a brain neoplasm. On the other hand, the definitive diagnosis had yet to be determined.

This case highlights the diagnostic challenges associated with CNS lesions and the necessity of maintaining a broad differential diagnosis. It shows the pivotal role of incorporating epidemiological data into diagnostic reasoning. In this instance, the patient’s origin from a region with a high prevalence of HIV and tuberculosis should have prompted earlier consideration of opportunistic infections.

The evaluation of CNS lesions in HIV-positive patients requires careful consideration of the level of immunosuppression, typically reflected by the CD4+ count, as this profoundly influences the differential diagnosis. In patients with CD4+ counts below 200 cells/μL, opportunistic infections such as TE, PCNSL, and tuberculomas dominate the differential, along with progressive multifocal leukoencephalopathy (PML) [11].

This patient’s presentation of multiple ring-enhancing lesions with significant edema and mass effect was consistent with TE. However, the differential also included PCNSL, metastatic lesions, and tuberculomas, particularly given her geographic origin.

While imaging findings are important in narrowing the differential diagnosis, they are often nonspecific. TE typically presents with multiple ring-enhancing lesions, whereas PCNSL may appear as solitary or irregularly enhancing lesions with periventricular involvement. Tuberculomas may also present with ring enhancement, often affecting the basal ganglia, and must be considered in patients from endemic areas. Moreover, PML lesions are generally non-enhancing but can enhance and cause mass effects in the context of immune reconstitution inflammatory syndrome [1,12].

In this case, the patient’s severe immunosuppression, positive *T. gondii* serology, and radiologic improvement following empiric TE therapy strongly supported the diagnosis [4,6], later confirmed with the detection of toxoplasma DNA in CSF. However, the absence of diagnostic specificity in imaging and initial tests underscores the importance of combining epidemiological, clinical, and laboratory data to guide management.

Empiric treatment for TE is a fundamental cornerstone in managing patients with characteristic radiological findings and advanced HIV [6]. The patient’s clinical improvement and radiological response confirmed the diagnosis and obviated the need for more invasive procedures.

Corticosteroids, used judiciously in this case to manage cerebral edema, illustrate the dual-edged nature of their role in CNS lesion management. While they are essential for preventing herniation in cases of significant mass effect, their use remains controversial in TE. The role of adjunct corticosteroids in TE is unclear, as highlighted by a systematic review where corticosteroids were used in 16.9%-90.2% of patients across 10 studies. Subgroup analyses from three of these studies found no significant differences in outcomes or safety associated with corticosteroid treatment [13]. Furthermore, corticosteroids can obscure diagnostic imaging and interfere with biopsy interpretation, particularly in cases of PCNSL, further complicating their application in CNS lesion management [14].

The importance of maintaining a broad diagnostic perspective cannot be overstated. Anchoring bias, particularly in settings with limited initial data, may lead to premature conclusions, overlooking treatable conditions. This patient’s journey - from a presumed malignancy to a treatable infection - emphasizes the transformative potential of timely, accurate diagnoses in advanced HIV.

## Conclusions

Cerebral toxoplasmosis can present indistinguishably from primary brain neoplasms in patients with undiagnosed immunocompromise. Integrating thorough clinical and epidemiological assessments while keeping a broad diagnostic perspective is of utmost importance. Sustaining supportive care and avoiding premature therapeutic limitations, even when initial presentations are misleading, facilitated a robust diagnostic process, enabling targeted treatment. In this case report, the patient’s trajectory, from presenting with an initial diagnosis of brain neoplasm and being considered for early palliative care to being diagnosed with a treatable infectious disease, culminated in sufficient recovery to be discharged home. The key message is the importance of maintaining support until a definitive diagnosis is reached, avoiding early irreversible decisions.
